# Loss of Atrx Affects Trophoblast Development and the Pattern of X-Inactivation in Extraembryonic Tissues

**DOI:** 10.1371/journal.pgen.0020058

**Published:** 2006-04-21

**Authors:** David Garrick, Jackie A Sharpe, Ruth Arkell, Lorraine Dobbie, Andrew J. H Smith, William G Wood, Douglas R Higgs, Richard J Gibbons

**Affiliations:** 1 MRC Molecular Haematology Unit, Weatherall Institute of Molecular Medicine, University of Oxford, Oxford, United Kingdom; 2 MRC Mammalian Genetics Unit, Harwell, Oxfordshire, United Kingdom; 3 Institute for Stem Cell Research, University of Edinburgh, Edinburgh, United Kingdom; The Babraham Institute, United Kingdom

## Abstract

ATRX is an X-encoded member of the SNF2 family of ATPase/helicase proteins thought to regulate gene expression by modifying chromatin at target loci. Mutations in *ATRX* provided the first example of a human genetic disease associated with defects in such proteins. To better understand the role of ATRX in development and the associated abnormalities in the ATR-X (alpha thalassemia mental retardation, X-linked) syndrome, we conditionally inactivated the homolog in mice, *Atrx,* at the 8- to 16-cell stage of development. The protein, Atrx, was ubiquitously expressed, and male embryos null for Atrx implanted and gastrulated normally but did not survive beyond 9.5 days postcoitus due to a defect in formation of the extraembryonic trophoblast, one of the first terminally differentiated lineages in the developing embryo. Carrier female mice that inherit a maternal null allele should be affected, since the paternal X chromosome is normally inactivated in extraembryonic tissues. Surprisingly, however, some carrier females established a normal placenta and appeared to escape the usual pattern of imprinted X-inactivation in these tissues. Together these findings demonstrate an unexpected, specific, and essential role for Atrx in the development of the murine trophoblast and present an example of escape from imprinted X chromosome inactivation.

## Synopsis

ATRX belongs to a class of proteins that may modify how DNA is packaged into chromatin, altering the accessibility of other proteins in the nucleus to DNA. In this way, ATRX is thought to influence gene expression. Mutations in the *ATRX* gene, which is located on the female sex chromosome (X), provided the first example of a human disease (ATR-X syndrome) associated with defects in such proteins. Affected males (X^MUT^Y) have multiple developmental abnormalities in a wide variety of systems. Currently, it is not understood how proteins like ATRX influence cell biology. To address this question, the authors deleted the version of the gene in mice, *Atrx*. Although affected male mice (X^MUT^Y) started to develop normally, they died early in development because they failed to form a normal placenta. In the placenta, female mice normally inactivate the X chromosome that they inherit from their fathers (Xp), so if females inherit from their mother an X chromosome (Xm) that bears the abnormal copy of *Atrx* (Xm^MUT^Xp), one would predict that, like affected males, they would fail to form a normal placenta. The authors unexpectedly found this not to be so. They showed, instead, that in such females the normal, paternally derived *Atrx* gene is active. This study has therefore demonstrated an important facet of X-chromosome imprinting.

## Introduction

ATR-X syndrome is a severe, nonprogressive form of X-linked mental retardation that is frequently associated with multiple congenital abnormalities [1]. It is usually associated with a mild form of α-thalassaemia, caused by reduced expression of structurally intact α-globin genes, and characterised by the presence of β-globin tetramers (haemoglobin H inclusion bodies) in peripheral red blood cells. Carrier females occasionally manifest haemoglobin H inclusions, but are otherwise intellectually and physically normal. Studies of X-chromosome inactivation in carrier females have demonstrated preferential inactivation of the chromosome bearing the abnormal allele in a variety of tissues [2], and this skewing of X-inactivation is thought to explain the mild phenotype observed in carriers.

**Figure 1:**
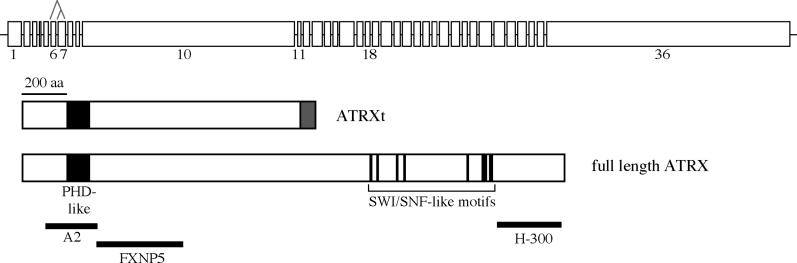
Schematic Representation of the ATRX Isoforms Shown at the top is the human *ATRX* cDNA. The boxes represent the 36 exons. The introns are not to scale. The alternative splicing of exons 6 and 7 is indicated. Shown underneath are the two ATRX protein isoforms. Full-length ATRX (~280 kDa) is encoded by the largest open reading frame. The positions of the principal features (the PHD-like domain and the seven SWI/SNF-like motifs) are indicated. Above full-length ATRX is shown the truncated ATRXt isoform (apparent molecular weight of ~200 kDa) that arises through the failure to splice intron 11 and the use of an intronic poly(A) signal. The intron-encoded region of ATRXt is indicated as a filled grey box. Locations of recombinant proteins (A2, FXNP5, and H-300) used to generate antibodies are shown. The scale bar represents 200 amino acids.

The ATR-X syndrome is caused by mutations in a gene *(ATRX)* that comprises 36 exons spanning 300 kb of genomic DNA at Chromosome Xq13.3 [3]. This gene encodes two dominant protein isoforms (Figure 1). As well as the full-length ATRX protein of ~280 kDa, which is encoded by a transcript of ~10 kb, we recently demonstrated that a truncated isoform called ATRXt (~200 kDa) is produced from a transcript of around 7 kb, which arises when intron 11 fails to be spliced from the primary transcript and an alternative intronic poly(A) signal is used [4]. The mouse homolog of the *ATRX* gene, *Atrx,* is also situated on the X chromosome, and also gives rise to full-length (Atrx, ~280 kDa) and truncated (Atrxt, ~200 kDa) isoforms [4,5].

Disease-causing missense mutations are clustered in two regions of the gene: a PHD-like zinc finger domain and a SNF2-like ATPase domain (Figure 1) [6]. The former motif is thought to be involved in protein-protein interactions in chromatin [7], and the latter is a feature of chromatin-remodelling proteins, and the presence of disease-causing mutations indicates the functional importance of these domains. ATRX has been shown to remodel chromatin [8]. It also interacts with HP1 at heterochromatin [9] and is recruited to promyelocytic leukemia nuclear bodies via an interaction with Daxx [10]. Furthermore, disruption of ATRX leads to diverse changes in DNA methylation [11]. Nevertheless, the role ATRX plays in gene expression remains unclear.

The consistent core of clinical and haematological features observed in ATR-X patients suggests that, like the SWI2/SNF2 chromatin-remodelling protein, ATRX probably regulates transcription of a discrete set of target genes. However, although there are clearly others to be found, at present the α-globin genes remain the only confirmed targets for transcriptional regulation by ATRX. Little is currently known about the precise role of the ATRX protein during mammalian development. To investigate the role of this protein during mouse development, we generated a conditionally deleted allele of the *Atrx* gene in mouse embryonic stem (ES) cells, and used these cells to examine the effect of ablating expression of the full-length Atrx protein in ES cells and in mouse embryos.

## Results

### Generation of ES Cells Lacking Full-Length Atrx

**Figure 2:**
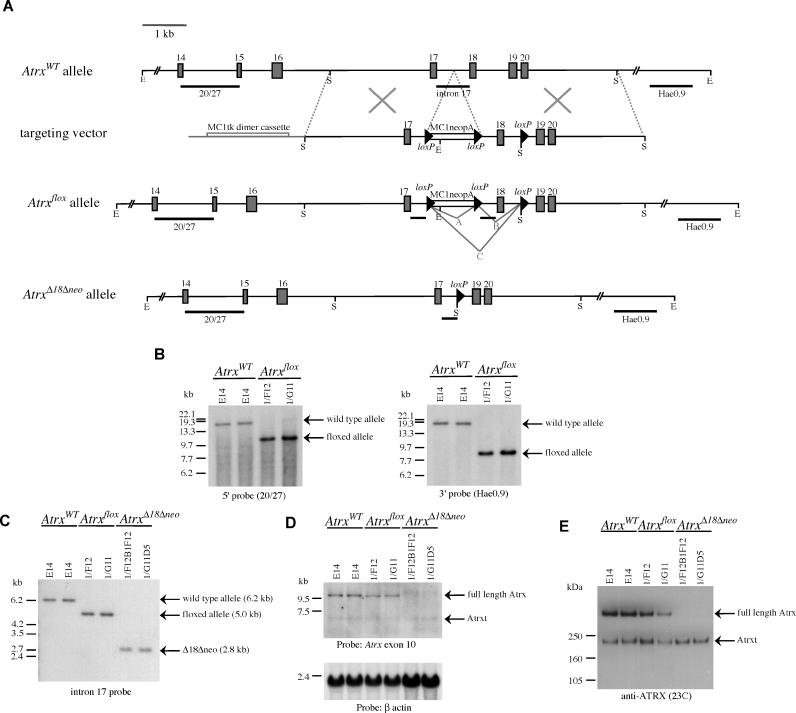
Cre-Mediated Ablation of Full-Length Atrx Protein in ES Cells (A) Strategy for targeted deletion of exon 18 of the *Atrx* gene. The top line shows the wild-type allele (*Atrx* ^WT^) at the region surrounding exon 18. Below is shown the targeting vector and the targeted allele (*Atrx* ^flox^) resulting from homologous recombination. The *loxP* target sites of the Cre recombinase are shown as black triangles, and the three possible recombination events that can be mediated by the Cre recombinase are indicated (labelled A, B, and C in the *Atrx* ^flox^ allele). At bottom is shown the Cre-recombined allele (*Atrx* ^Δ18Δneo^) (resulting from recombination event C) in which both exon 18 and the MC1neopA selection cassette have been deleted. EcoRI (labelled E) and SacI (labelled S) sites present on the targeted 129 strain X chromosome are indicated. Black bars indicate the positions of the probes used in Southern blots. (B) Southern blot analysis of EcoRI-digested DNA from either wild-type ES cells (E14) or targeted ES cell clones bearing the *Atrx* ^flox^ allele (1/F12 and 1/G11) hybridised with either the 20/27 (left blot) or Hae0.9 (right blot) probes. The EcoRI fragment of the *Atrx* ^WT^ allele (18.5 kb) has been replaced with the expected fragments of 11.2 kb (20/27 probe) or 8.5 kb (Hae0.9 probe) (C) Southern blot analysis of SacI-digested DNA from either wild-type ES cells (E14) or targeted ES cell clones bearing the *Atrx* ^flox^ allele (1/F12 and 1/G11) or Cre-recombinant clones derived from these (1/F12B1F12 and 1/G11D5). The membrane was hybridised with the intron 17 probe indicated in (A). The expected bands of 6.2 (*Atrx* ^WT^), 5.0 (*Atrx* ^flox^), and 2.8 (*Atrx* ^Δ18Δneo^) kb were observed. (D) Northern blot analysis of RNA from ES cells shown in (C). The membrane was hybridised first to a probe from exon 10 of the *Atrx* gene (top blot) and subsequently to a β-actin cDNA probe as loading control (bottom blot). The transcripts responsible for full-length Atrx (~10 kb) and the truncated Atrxt isoforms (~7 kb) are indicated. (E) Western blot analysis of whole-cell extracts from the clones shown in (C) using an anti-ATRX monoclonal antibody (23C, raised against peptide A2 of the human ATRX protein shown in Figure 1). The full-length and truncated Atrx isoforms are indicated.

Like the human gene, the mouse *Atrx* gene is also X-linked, such that a direct disruption of the single *Atrx* allele in male ES cells would immediately give rise to the null state. No targeted clones were recovered after attempted homologous recombination in male E14TG2a ES cells using two different vectors that removed exon 18 of the *Atrx* gene. Exon 18 encodes the first of the seven motifs composing the conserved SNF2-like domain of Atrx (Figure 1); mutation of the corresponding motif of the yeast SNF2 protein has been shown to severely impair SWI/SNF-dependent gene expression [12]. The failure to recover targeted clones with these vectors suggested that Atrx may be important for normal ES cell growth and expansion and that direct targeting of the single locus may not be possible. We therefore adopted a conditional strategy for targeting exon 18 (Figure 2) and recovered two clones in which exon 18 has been flanked by *loxP* recognition sites for the Cre recombinase (*Atrx* ^flox^ allele in Figure 2A) (Figure 2B). This allele also contains a *loxP*-flanked MC1-neo^r^ cassette in intron 17 (Figure 2A). Northern and Western blot analyses (Figure 2D and 2E) confirmed that the *Atrx* ^flox^ clones continued to express both full-length Atrx protein and the truncated Atrxt isoform.

To generate the full deletion in ES cells, the *Atrx* ^flox^ clones (1/F12 and 1/G11) were transiently transfected with a Cre-recombinase expression plasmid (pCAGGS-Cre-IRES*puro*), and subclones were recovered bearing an allele (*Atrx* ^Δ18Δneo^ in Figure 2A) in which both exon 18 and the neo^r^ cassette had been deleted by the Cre recombinase (resulting from the recombination event labelled “C” in the *Atrx* ^flox^ allele shown in Figure 2A) (Figure 2C). Northern and Western blot analyses (Figure 2D and 2E) revealed that the full-length Atrx transcript and protein is completely abolished in the *Atrx* ^Δ18Δneo^ recombinant clones, suggesting that deletion of this region has a highly destabilising effect on the full-length transcript. As expected, the truncated Atrxt isoform, the transcript of which is terminated within intron 11 [4], is unaffected by the deletion of exon 18 (Figure 2E). While the function of Atrxt is not yet clear, this isoform, which contains the PHD-like domain but not the SWI/SNF motifs (Figure 1), is unlikely to be functionally equivalent to the full-length protein. Thus, a conditional knockout strategy allowed the isolation of ES cells that are null for full-length Atrx.

### Perturbed Growth and Methylation Defects in Atrx^null^ ES Cells

**Figure 3:**
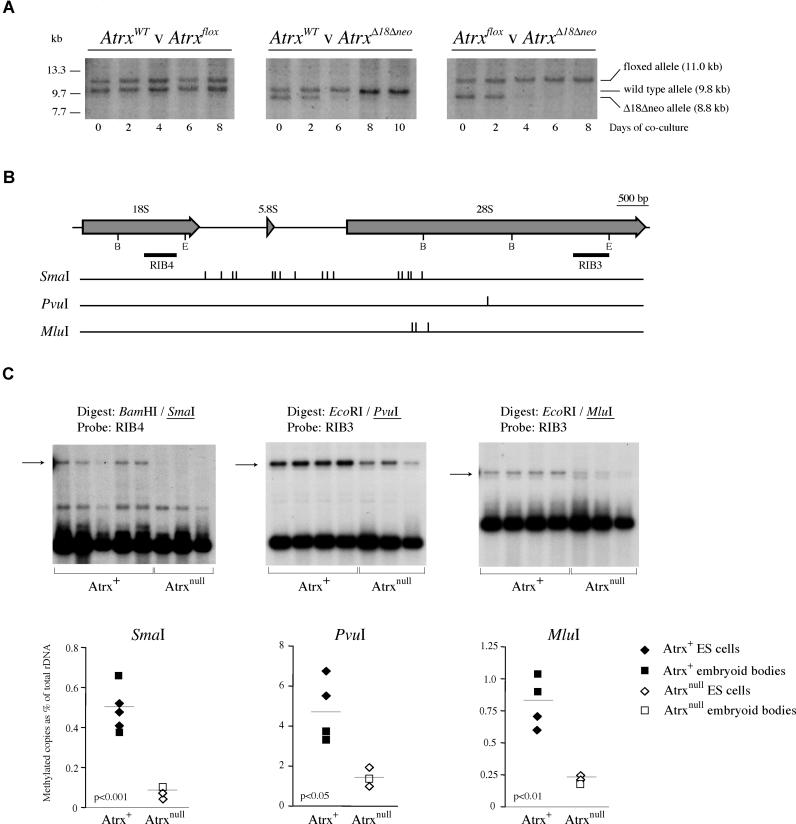
Growth and Methylation Defects in Atrx^null^ ES Cells (A) Cultures were inoculated with equivalent numbers of ES cells bearing different *Atrx* alleles as indicated, and were serially passaged. After the indicated days of coculture, DNA extracted from a sample of cells was analysed by Southern blot to detect the *Atrx* alleles. DNA was digested with SpeI, and the membrane was hybridised with the 20/27 probe shown in Figure 2A. The expected sizes of the different alleles are indicated. (B) Schematic diagram of the transcribed portion of the mouse rDNA repeat with the 18S, 5.8S, and 28S genes indicated. The positions of the limit-digesting enzymes BamH (labelled B) and EcoRI (labelled E) and the probes (RIB3 and RIB4) used in the Southern blots shown in (C) are indicated. Below are shown the locations of the methylation-sensitive enzymes (SmaI, PvuI, and MluI) whose methylation status has been analysed in the Southern blots shown in (C). (C) DNA from Atrx-positive (Atrx^+^, bearing either an *Atrx* ^WT^ or *Atrx* ^flox^ allele) or Atrx^null^ (bearing the *Atrx* ^Δ18Δneo^ allele) ES cells and 7-d embryoid bodies were digested with the enzymes shown and analysed by Southern blotting using the probes indicated. Arrows indicate the fully methylated copies (cut by only the limit-digesting enzyme). Phosphorimager quantitation of the blots are shown below. The *y*-axis shows the percentage of copies that are undigested by the methylation-sensitive enzyme as a percentage of the total signal from cut and uncut rDNA. Mean values are indicated by horizontal lines, and the significance of the differences between the Atrx-positive and Atrx^null^ populations are shown for each enzyme.

Atrx^null^ ES cells could be maintained in culture but were generally slower growing than Atrx^+^ ES clones, and appeared to undergo higher rates of spontaneous differentiation. We investigated directly the effect of Atrx on ES cell growth by comparing Atrx^+^ and Atrx^null^ ES cell clones in competition cultures. Equal numbers of Atrx^+^ (bearing either an *Atrx* ^WT^ or an *Atrx* ^flox^ allele) and Atrx^null^ (bearing an *Atrx* ^Δ18Δneo^ allele) ES cells were inoculated into cultures and the mixed cultures were passaged (1:3 split) every 2 d for 8–10 d. The relative abundance of the different alleles in the culture at each time point was analysed by Southern blotting (Figure 3A). The clone containing the *Atrx* ^Δ18Δneo^ allele was rapidly outgrown by both *Atrx* ^WT^ ES cells and cells bearing the *Atrx* ^flox^ allele. In a control competition experiment between different clones bearing functional *Atrx* alleles (*Atrx* ^WT^ and *Atrx* ^flox^), both clones continued to be equally represented after 8 d of cocultivation. Thus, although Atrx^null^ ES cells could be recovered and maintained in culture by a conditional targeting approach, these cocultivation experiments suggested that the absence of Atrx does negatively impact upon normal ES cell growth.

To investigate a possible cell-cycle defect in the absence of Atrx, we analysed the cell cycle distribution of bromodeoxyuridine (BrdU)-pulsed ES cells by flow cytometry (Figure S1A). Surprisingly, both Atrx^null^ ES cell clones exhibited a cell cycle profile that was indistinguishable from ES cells bearing a functional *Atrx* allele (*Atrx* ^WT^ or *Atrx* ^flox^). We also specifically quantitated the mitotic index within each population by flow cytometry after staining ES cells for phosphorylated (Ser10) histone H3, a specific marker of mitosis (Figure S1B) [13]. Consistent with the normal cell-cycle profile observed above, there was no depletion in the size of the mitotic population in the Atrx^null^ ES clones, despite their slow growth. Finally, we investigated whether the growth defect in the Atrx^null^ ES cells was due to an up-regulation of apoptosis by staining cells with Annexin V (Figure S2) and found that the proportion of apoptotic cells was not significantly affected by the absence of full-length Atrx. Thus, the growth defect observed in ES cells lacking Atrx is not due to a specific cell cycle block or significant induction of cell death. While the cause of the proliferative delay is not yet clear, since Atrx^null^ ES cells appear to undergo higher rates of spontaneous differentiation (unpublished data), it seems likely that the observed growth defect reflects the spontaneous transition from fast-cycling, undifferentiated ES cells into more slowly cycling, differentiated cell types in these cultures.

It has been shown that disease-causing mutations in the human *ATRX* gene give rise to changes in the normal pattern of DNA methylation at several repetitive sequences within the human genome [11]. Notably, the transcribed region of the ribosomal DNA (rDNA) repeat was found to be significantly hypomethylated in ATR-X patients relative to normal individuals. Using methylation-sensitive restriction enzymes, we also observed significant hypomethylation at several sites tested within the mouse rDNA repeats in Atrx^null^ ES cells and 12-d embryoid bodies relative to ES cells and embryoid bodies bearing a functional *Atrx* allele (*Atrx* ^WT^ or *Atrx* ^flox^) (Figure 3B and 3C). The observation that rDNA is hypomethylated in the absence of Atrx, even in ES cells, is consistent with the finding that hypomethylation of the human rDNA repeats is detectable from an early developmental stage in ATR-X patients. Other mouse repetitive sequence elements surveyed in ES cell DNA include the heterochromatic major satellite (assayed with MaeII) and minor satellite (assayed with HpaII) repeats, as well as interspersed retroviral repeats of the intracisternal A particle (IAP) type and the Line 1 and Sine B1 families (all assayed with HpaII). These repeats were found to be moderately (Line 1 and Sine B1) or highly (IAP, major satellite, minor satellite) methylated in wild-type ES cells, and this methylation was not detectably perturbed by the absence of Atrx (Figure S3 and unpublished data). Taken together, these data indicate that the subtle interplay between the ATRX protein and DNA methylation observed in human patients is also present in mouse cells.

### Early Embryonic Lethality in Atrx^null^ Male Mice

**Figure 4:**
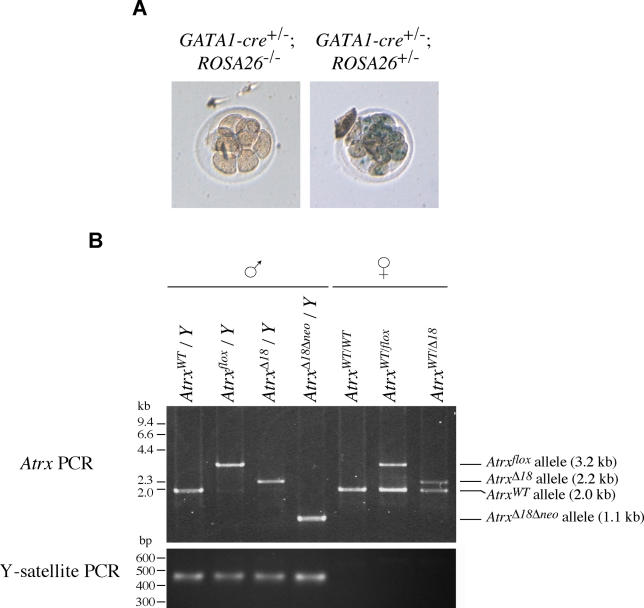
Timing of Onset of *GATA1-Cre* Expression and PCR Genotyping of *Atrx* Alleles (A) *GATA1-cre* ^+*/*+^ transgenic males were crossed to females of the ROSA26 reporter strain (*ROSA26* ^+*/*−^), and embryos were recovered at 0.5 dpc (~16-cell morula stage) and stained with X-gal. Cre-mediated activation of the *ROSA26* β-galactosidase reporter allele was detected in all cells in embryos in which both alleles are coinherited. (B) Top gel: PCR genotyping of *Atrx* alleles in embryos using primers PPS1.15 (exon 17) and Mxnp30 (exon 20) as described in Protocol S1. The sizes of PCR products from the different alleles are indicated. Both the *Atrx* ^Δ18^ (resulting from recombination event B in Figure 2A) and the *Atrx* ^Δ18Δneo^ allele (resulting from recombination event C in Figure 2A) are null for full-length Atrx protein. The bottom gel shows products from a PCR reaction (primers DG52/DG53) used to sex embryos as described in Protocol S1. A 450-bp PCR product is amplified from a mouse Y chromosome-specific satellite repeat.

To investigate the role of the Atrx protein during mouse development, we initially established lines of mice bearing the *Atrx* ^flox^ allele. Two independently targeted *Atrx* ^flox^ ES cell clones with normal male karyotype were injected into C57BL/6 blastocysts to produce chimaeric mice, which were then used to obtain germline transmission. Intercrosses between males hemizygous (*Atrx* ^flox^/Y) and females heterozygous (*Atrx* ^WT/flox^) for the floxed allele were also carried out to generate homozygous females (*Atrx* ^flox/flox^). Males hemizygous and females heterozygous or homozygous for the *Atrx* ^flox^ allele were viable, appeared healthy, and bred normally, suggesting that, as expected, the *Atrx* ^flox^ allele was functionally normal. To generate Atrx^null^ mice by Cre-mediated recombination, the *Atrx* ^flox^ mice were crossed with mice harboring a transgene in which the Cre recombinase is expressed under the control of the regulatory elements of the mouse *GATA-1* gene *(GATA1-cre)* [14]. Widespread expression of the *GATA1-cre* transgene has been demonstrated during early embryogenesis [14]. We more accurately defined the onset of *GATA1-cre* expression using a ROSA26 reporter strain, in which a β-galactosidase/neo^r^ fusion reporter gene is expressed only after Cre-mediated excision of *loxP*-flanked transcription and translation termination signals [14]. We found that the *GATA1-cre* transgene was already active at the 16-cell morula stage of development (0.5 days postcoitus [dpc]) (Figure 4A).

To generate Atrx^null^ mice, heterozygous floxed females (*Atrx* ^WT/flox^) were mated with homozygous *GATA1-cre* transgenic males *(Atrx* ^WT^/Y;*GATA1-cre* ^+*/*+^ *)*. No Atrx^null^ males *(Atrx* ^null^/Y;*GATA1-cre* ^+*/*−^ *)* were recovered at birth, indicating that the absence of Atrx results in embryonic lethality. This finding was unexpected, since human ATR-X patients clearly survive to adulthood (see Discussion). Embryos were dissected at 7.5, 8.5, and 9.5 dpc and genotyped by PCR analysis of DNA extracted from yolk sac or total embryo (Figure 4B and Protocol S1). Atrx^null^ males were present at expected mendelian ratios (~25%) at both 7.5 dpc and 8.5 dpc (Table 1). However, by 9.5 dpc, depletion was observed both in the number of Atrx^null^ males (7%) and in the total number of males recovered (31%). No Atrx^null^ males were recovered after 9.5 dpc. Thus the absence of Atrx gives rise to embryonic lethality in mice before 9.5 dpc.

**Table 1:**
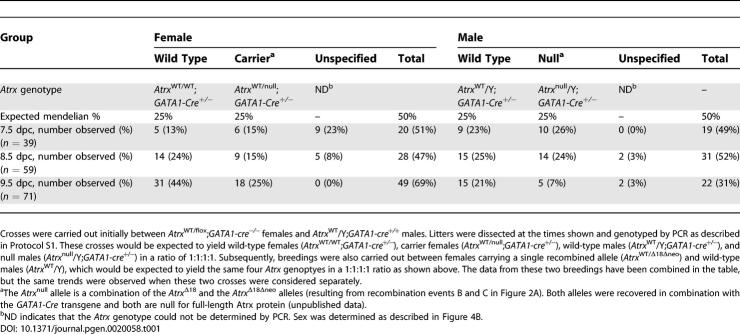
Distribution of *Atrx* Genotypes in Timed Matings

**Figure 5:**
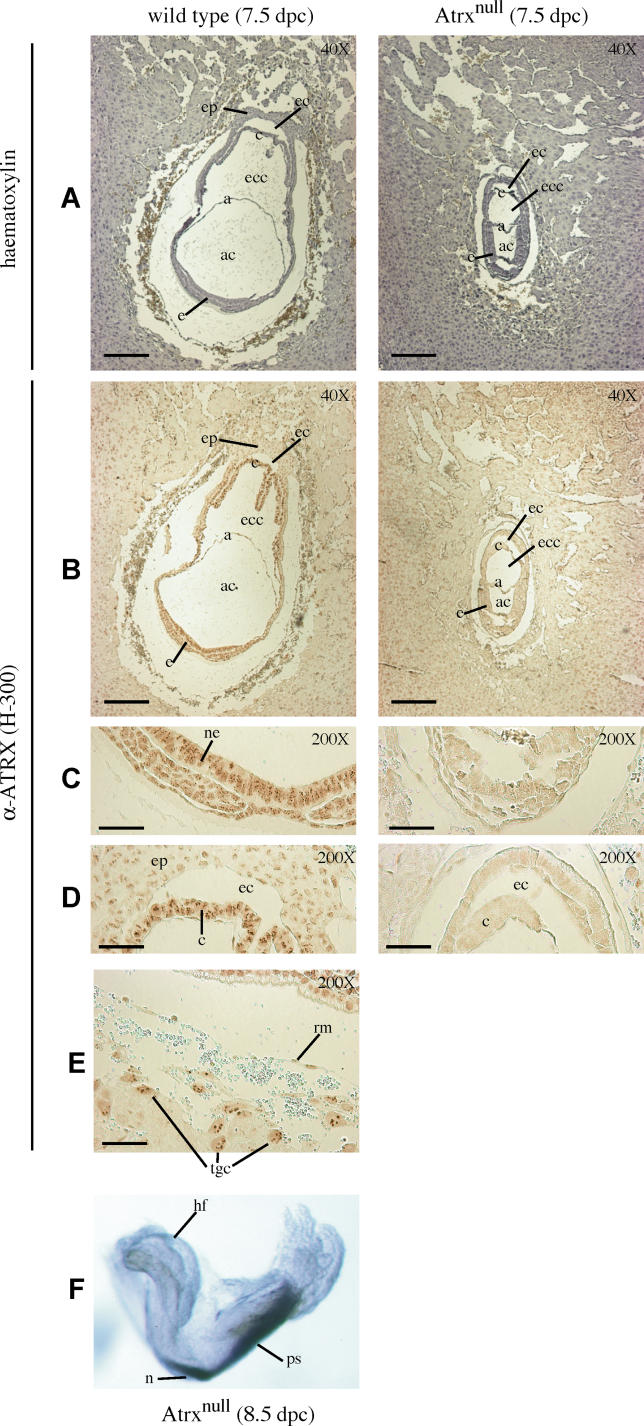
Morphology of Atrx^null^ Embryos at 7.5 dpc and 8.5 dpc Paraffin sections of wild-type or Atrx^null^ 7.5 dpc embryos (dissected in their deciduas) were stained with haematoxylin (A) or with an anti-ATRX antibody (H-300, Figure 1) (B–E). Photomicrographs C–E show higher magnification images (200×) of the stained sections shown in (B) (40×). Scale bars represent 200 μm (40× magnification) or 40 μm (200× magnification). a, amnion; ac, amniotic cavity; c, chorion; e, epiblast; ec, ectoplacental cavity; ecc, exocoelomic cavity; ep, ectoplacental cone; ne, neural ectoderm; rm, Reichert's membrane; tgc, trophoblast giant cell. (F) Detection of *brachyury (T)* expression in Atrx^null^ 8.5 dpc embryo (head fold stage) by WMISH. The genotype was determined by PCR (as shown in Protocol S1) using DNA extracted from yolk sac. hf, head fold; n, emerging notochord; ps, primitive streak.

To investigate the morphology of Atrx^null^ embryos prior to death, embryos from the above crosses were initially dissected in their deciduas at 7.5 dpc, and paraffin sections were stained with haematoxylin (Figure 5A) or with an anti-ATRX antibody (Figure 5B–5E). Immunohistochemical staining revealed that Atrx was widely expressed in wild-type 7.5 dpc embryos (Figure 5B). Expression was highest in the embryonic region (Figure 5C) and the chorion (Figure 5D). Detectable but lower levels of expression were observed in the ectoplacental cone (Figure 5D) and surrounding decidual tissue. We also observed very high levels of Atrx expression in trophoblast giant cells (TGCs) surrounding the Reichert's membrane (Figure 5E). Within the large nuclei of these TGCs, the typical nuclear association of Atrx with blocks of pericentromeric heterochromatin [15] was clearly observable. Only background staining was seen in the corresponding Atrx^null^ embryonic tissues (Figure 5B–5D), while expression in the surrounding decidual tissue (of maternal origin) was normal and served as an antibody staining control (unpublished data). Morphologically, 7.5 dpc Atrx^null^ embryos were dramatically reduced in size and appeared developmentally retarded relative to stage-matched wild-type embryos (Figure 5A and 5B). However, despite their reduced size, the general morphology and organisation of embryonic structures in Atrx^null^ conceptuses appeared grossly normal. The amnion and chorion were clearly present and the amniotic, exocoelomic, and ectoplacental cavities were distinguishable, as were all three embryonic germ layers (Figure 5A–5C). At 8.5 dpc, embryos were dissected free of deciduas, and observed in whole mount. Individual conceptuses were genotyped by PCR using DNA isolated from yolk sac as described in Protocol S1. Consistent with observations at 7.5 dpc, the general morphology of the embryo proper of Atrx^null^ conceptuses also appeared grossly normal at 8.5 dpc. The head fold had clearly formed, and expression of the early mesoderm marker *brachyury (T)* [16] was detected in the primitive streak and emerging notochord by whole-mount in situ hybridisation (WMISH) (Figure 5F), indicating that Atrx^null^ embryos had gastrulated.

**Figure 6:**
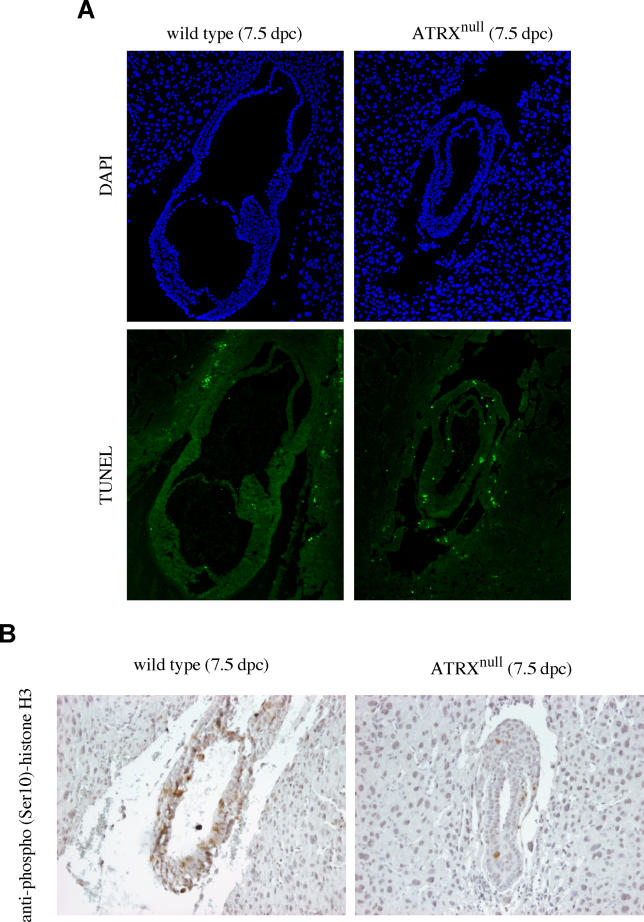
Analysis of Apoptosis and Mitosis in Atrx^null^ Embryos (A) Paraffin sections of wild-type or Atrx^null^ 7.5 dpc embryos (dissected in their deciduas) were analysed by TUNEL assay and apoptotic cells labelled with fluorescein-dUTP. Sections were counterstained with DAPI. (B) Paraffin sections of wild-type or Atrx^null^ 7.5 dpc embryos were stained with an antibody against the mitosis marker phosphorylated (Ser10) histone H3. Sections were counterstained with haematoxylin. For both (A) and (B), the presence or absence of Atrx in each embryo was determined by staining adjacent sections with the anti-ATRX antibody (H-300) as in Figure 5 (unpublished data).

To investigate whether the reduced size of the Atrx^null^ embryos was due to an increase in apoptosis, we analysed sections of paraffin-embedded 7.5 dpc embryos by TdT-mediated dUTP nick end labeling (TUNEL) assay (Figure 6A). Very few apoptotic cells were detected in wild-type 7.5 dpc embryos. In Atrx^null^ embryos, a slight increase in the apoptotic population was evident. However, consistent with our observation of a grossly normal apoptotic index in Atrx^null^ ES cells (Figure S2), the apoptotic response observed in Atrx^null^ embryos was also not uniform, but was restricted to a low number of scattered TUNEL-positive cells. Since this small apoptotic response is unlikely to account fully for the dramatic size deficit observed in Atrx^null^ embryos, a possible proliferation defect was also investigated by immunohistochemical staining of 7.5 dpc embryo sections for the mitosis marker phosphorylated (Ser10) histone H3 [13]. Relative to the very high mitotic index observed in wild-type embryos, the proportion of mitotic cells observed in Atrx^null^ embryos at 7.5 dpc was dramatically reduced (Figure 6B). Taken together, these results suggest that the size deficit observed in Atrx^null^ embryos prior to lethality largely reflects a proliferative defect, with a minor but indirect contribution from increased apoptosis. Although a growth defect was also observed in Atrx^null^ ES cells (Figure 3A), in contrast to the Atrx^null^ embryos, the mitotic index of the ES cell population (as measured with the same antibody) was not depleted (Figure S1B). These observations suggest that the mitotic defect observed in embryos is unlikely to be a direct, cell-autonomous effect of the absence of Atrx, and is more likely to be a secondary effect resulting from the failure to develop a normal trophoblast (see below).

### Trophectoderm Failure in Atrx^null^ Embryos

**Figure 7:**
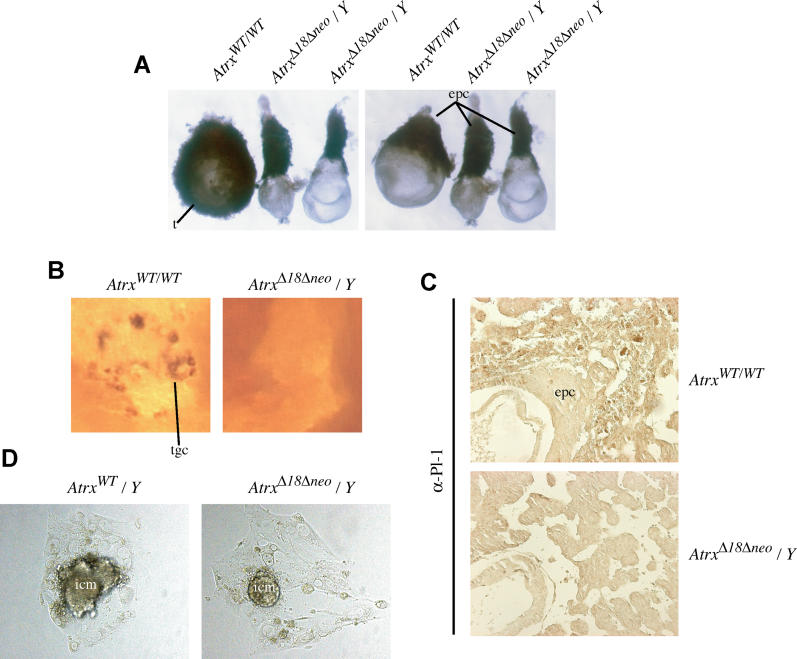
Trophectoderm Defect in Atrx^null^ Embryos (A) 8.5 dpc embryos were dissected from surrounding decidual tissue and observed in whole mount. The genotype of each (indicated above) was determined by PCR using DNA extracted from whole embryos after photography. In the left image, the wild-type female (three-somite stage, left) is surrounded by trophoblast (t) while the trophoblast component surrounding the Atrx^null^ males (at headfold/presomite [middle] and two-somite stages [right], respectively) is severely depleted. In the right image, the trophoblast has been dissected away from the embryonic region of the wild-type embryo, to reveal the small, abnormally shaped ectoplacental cone (epc) of the mutant littermates. (B) WMISH to detect expression of *Pl-1* (a marker of TGCs) at the implantation sites in vacated deciduas that had contained 8.5 dpc wild-type (*Atrx* ^WT/WT^) or Atrx^null^ (*Atrx* ^Δ18Δneo^/Y) embryos. The genotype was determined by PCR using DNA extracted from whole embryos. TGCs are stained with Pl-1. (C) Paraffin sections of wild-type or Atrx^null^ 7.5 dpc embryos (dissected in their deciduas) were stained with an anti-Pl-1 antibody. The presence or absence of Atrx in each embryo was determined by staining adjacent sections with the anti-ATRX antibody (H-300) as in Figure 5 (unpublished data). (D) Examples of 5-d blastocyst outgrowth cultures. Extensive trophoblast outgrowing from the inner cell mass (icm) was observed in all genotypes. The *Atrx* genotype and sex of the blastocyst indicated were determined by PCR.

Whole-mount observation of 8.5 dpc embryos revealed that, in contrast to the basically normal although delayed morphology of the embryo itself, the extraembryonic tissues of Atrx^null^ conceptuses appeared highly disorganised. When embryos were removed from deciduas, the surrounding trophectoderm layer appeared dramatically reduced in Atrx^null^ embryos relative to wild-type littermates, and the underlying ectoplacental cone appeared reduced and abnormally shaped (Figure 7A). Vacated deciduas surrounding 8.5 dpc wild-type and Atrx^null^ embryos were bisected and analysed by WMISH for expression of placental lactogen-1 (Pl-1)*,* a marker of terminally differentiated TGCs. The number of Pl-1-expressing cells attached to the decidual wall after removal of the embryo is an indication of the density of trophoblast cells surrounding each implantation site [17]. We found that the population of Pl-1-expressing cells was depleted in the decidual implantation sites containing Atrx^null^ embryos relative to those containing wild-type littermates (Figure 7B); this was also apparent at 7.5 dpc, as determined by immunohistochemical staining of paraffin sections of embryos in deciduas with an anti-Pl-1 antibody (Figure 7C). A TGC deficiency in the absence of Atrx is consistent with the observation that Atrx is highly expressed in giant cells surrounding wild-type 7.5 dpc embryos (Figure 5E).

To investigate whether the trophoblast defect was restricted to the production of secondary TGCs (produced by diploid precursors in the ectoplacental cone and derived originally from the polar trophectoderm overlying the inner cell mass of the blastocyst) or also affected the production of primary TGCs (resulting from differentiation of the mural trophectoderm of the blastocyst), blastocysts (3.5 dpc) from crosses between *Atrx* ^WT/flox^ females and *GATA1-Cre* homozygous transgenic males (*Atrx* ^WT^/Y;*GATA1-Cre* ^+*/*+^) were cultured in vitro for 5 d to monitor outgrowth of the primary trophoblast. After 5 d, individual blastocyst cultures were scored for the extent of primary trophoblast outgrowth, and the *Atrx* genotype and sex of the blastocyst were determined by PCR. Most blastocysts hatched from the zona pellucida within 24 h, and trophoblast cells spreading out from the inner cell mass could usually be detected within 48 h of culture. No difference was observed in the rate or extent of trophoblast outgrowth over 5 d of culture between Atrx^null^ blastocysts (*Atrx* ^null^/Y, *n* = 6) and blastocysts bearing an *Atrx* ^WT^ allele (*Atrx* ^WT/WT^, *n* = 6; *Atrx* ^WT/null^, *n* = 6; *Atrx* ^WT^/Y, *n* = 6) (examples shown in Figure 7D), suggesting that the defect specifically involves the secondary giant cell compartment. This is consistent with the observation that Atrx^null^ conceptuses implant successfully and survive to gastrulation. Taken together, these data suggest that loss of Atrx results in a defect in formation of the secondary trophoblast that is apparent from 7.5 dpc. Despite initiating normal organisation in the embryo proper, Atrx^null^ conceptuses exhibit a proliferative defect by 7.5 dpc and die by around 9.5 dpc, probably due to a nutritional deficit resulting from failure to develop a normal trophoblast.

### Escape from Imprinted Inactivation of the Paternally Inherited *Atrx* ^WT^ Allele in Extraembryonic Tissues of Carrier Female Mice

Female mice carrying an *Atrx* ^null^ allele (*Atrx* ^WT/null^;*GATA1-cre* ^+*/*−^) were detected at 9.5 dpc (Table 1) and recovered at birth (unpublished data), although at both time points the number of carrier females was lower than that of wild-type (*Atrx* ^WT/WT^;*GATA1-cre* ^+*/*−^) females, suggesting that a proportion of carrier female embryos died in utero. Surviving adult carrier female mice were not phenotypically normal and exhibited mild behavioural abnormalities, although some could reproduce. For all *Atrx* ^WT/null^ carrier female embryos presented in Table 1, the *Atrx* ^WT^ allele was paternally derived, while the *Atrx* ^null^ allele was maternally derived. In the mouse, X chromosome inactivation is subject to parental imprinting in the trophectoderm and primitive endoderm lineages that give rise to the extraembryonic tissues, resulting in inactivation of the paternal X chromosome (Xp) [18]. In contrast, in tissues of the embryo proper (derived from the inner cell mass of the blastocyst) X-inactivation is random [19]. Thus, in the extraembryonic compartment of *Atrx* ^WT/null^ carrier females, normal imprinted X-inactivation would be expected to result in silencing of the paternally derived *Atrx* ^WT^ allele, leaving only the *Atrx* ^null^ allele on the active maternal X (Xm) and thereby render the extraembryonic tissues null for full-length Atrx protein. However, the absence of Atrx in the extraembryonic compartment is lethal in *Atrx* ^null^/Y males. This suggested the possibility of an escape from imprinted inactivation of the paternally derived *Atrx* ^WT^ allele in the extraembryonic compartment of a proportion of carrier female embryos.

**Figure 8:**
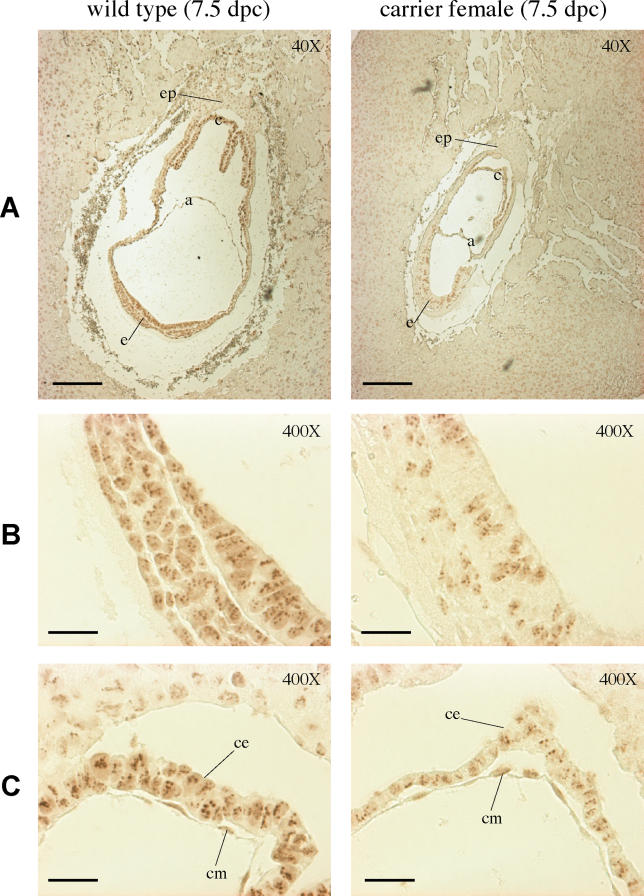
Escape from Imprinted Inactivation of the Paternally Inherited *Atrx^WT^* Allele in Carrier Females Paraffin sections of wild-type (*Atrx* ^WT^/Y) and carrier female (*Atrx* ^WT/null^) 7.5 dpc embryos (dissected in their deciduas) were stained with the anti-ATRX antibody (H-300). Scale bars represent 200 μm (40× magnification) or 20 μm (400× magnification). (A) Stained sections showing whole embryos at 40× magnification. a, amnion; c, chorion; e, epiblast; ep, ectoplacental cone. (B) Higher-magnification image (400×) of the epiblast regions of the stained sections shown in (A). (C) Higher-magnification image (400×) showing the extraembryonic derived-chorionic ectoderm of the stained sections shown in (A). ce, chorionic ectoderm; cm, chorionic mesoderm.

To investigate further, we crossed homozygous *Atrx* ^flox/flox^ females and homozygous *GATA1-cre* transgenic males (*Atrx* ^WT^/Y;*GATA1-cre* ^+*/*+^), which would be expected to yield only Atrx^null^ males (*Atr*x^null^/Y;*GATA1-cre* ^+*/*−^) and carrier females (*Atrx* ^WT/null^;*GATA1-cre* ^+*/*−^). In these carrier females, the *Atrx* ^WT^ allele is paternally inherited. Embryos were dissected in their deciduas at 7.5 dpc, and paraffin sections were stained with anti-ATRX antibody, along with sections from a wild-type 7.5 dpc embryo for comparison (Figure 8). As described above, Atrx expression was detected in every cell in the epiblast (embryo proper) region of wild-type 7.5 dpc embryos (Figure 8B). In contrast, the epiblast region of carrier female embryos was composed of a mosaic of small clusters of Atrx-positive cells (in which the *Atrx* ^null^ allele on Xm had been inactivated) and Atrx-negative cells (in which the *Atrx* ^WT^ allele on Xp had been inactivated), indicating that the *Atrx* gene was subject to normal random X-inactivation in the epiblast. Remarkably, clear Atrx expression could also be detected in the extraembryonic tissues of carrier females, as shown in the extraembryonic-derived chorionic ectoderm (Figure 8C). Atrx expression could be detected in almost all cells of the chorionic ectoderm. Atrx expression was also clearly detected in other extraembryonic structures, including TGCs (unpublished data). Escape from silencing of an Xp-inherited *Atrx* ^WT^ allele was also observed in the chorionic ectoderm of carrier females at 8.5 dpc (Figure S4). Thus, although random X-inactivation occurs normally within the epiblast, the *Atrx* ^WT^ allele (inherited on the Xp chromosome) escaped the normal imprinted X-inactivation in the extraembryonic compartment of some carrier females.

## Discussion

We investigated the role of the Atrx protein in mouse development. By using a conditional knockout approach, we ablated the full-length Atrx protein first in ES cells and embryoid bodies, and then in developing mouse embryos.

### Atrx in ES Cells

Atrx^null^ ES cells could not be recovered by direct targeting and were eventually generated by adopting a conditional targeting approach. This is consistent with our observation that Atrx is highly expressed in ES cells, and that the absence of full-length Atrx imparts a growth disadvantage relative to cells bearing a functional *Atrx* allele. At present, the cause of the proliferative delay in Atrx^null^ ES cells is not known. Interestingly, we demonstrated that apoptosis is not significantly up-regulated in ES cells lacking Atrx and is only mildly elevated in Atrx^null^ 7.5 dpc mouse embryos. In contrast, it was recently shown that the loss of Atrx markedly increased the apoptotic population in the differentiating cells of the embryonic cortex and postnatal hippocampus, when Atrx expression was ablated in the developing mouse forebrain using the *Atrx* ^flox^ allele described here [20]. The human ATRX protein has been shown to associate in a complex with Daxx [8], a protein that has been implicated in multiple pathways for the regulation of apoptosis [21]. It is possible that disruption of the mouse Atrx-Daxx complex (by ablation of the Atrx protein) could have triggered a universal proapoptotic response. However, our observations in ES cells demonstrate that the induction of apoptosis is not an automatic response triggered by the removal of Atrx in all cell types, and suggest that the inappropriate apoptosis observed in the *Atrx*-mutant forebrain may reflect a requirement for Atrx during terminal differentiation.

### An Unexpected Role for Atrx in Development of the Mouse Trophoblast

We show that Atrx^null^ male mice are not viable and embryos die by around 9.5 dpc. Before death, Atrx^null^ embryos exhibit a markedly reduced mitotic index, suggesting a proliferative defect. Although the embryonic compartment of the conceptus appears initially normal, by 7.5 dpc Atrx^null^ embryos display abnormalities in development of the trophoblast, including a depletion in the population of TGCs surrounding the conceptus and a reduction in the size of the ectoplacental cone, which contains the diploid giant cell precursors [22]. TGCs are highly differentiated, postmitotic cells that ultimately form an epithelial layer at the periphery of the placenta, which interfaces with the maternal cells of the decidua [23]. These highly invasive cells are important for mediating initial invasion of the uterine tissue, but are also involved in remodelling the maternal decidua after implantation and in secreting hormones that regulate fetal and maternal growth [24]. Since Atrx^null^ embryos appear to implant normally, lethality is likely to arise due to a failure of TGC function later during development.

Embryonic lethality in mice in the absence of Atrx was a surprising finding, as there had been no suggestion of foetal loss in the human ATR-X syndrome. It is possible that the role of Atrx in the trophoblast is specific to mice and that ATRX has no role or is redundant in the human trophoblast. Indeed, the birth weight of babies with ATR-X syndrome is usually normal. An alternative explanation for the unexpectedly severe phenotype we observed in mice is that the *Atrx* ^Δ18Δneo^ deletion generated by Cre recombination completely ablates full-length Atrx protein (Figure 2E). In contrast, all disease-causing mutations characterised in human ATR-X pedigrees appear to give rise to hypomorphic alleles. Some full-length ATRX protein is detected in cases predicted to have truncating mutations (RJG, unpublished data), and residual ATPase activity in ATRX immunoprecipitates can be detected in Epstein-Barr virus-transformed lymphocytes of all human patients analysed to date (A. Argentaro and M. Mitson, unpublished data). The failure to observe a truly null *ATRX* allele among human patients strongly suggests that, as in the mouse, the complete absence of ATRX protein is incompatible with human fetal survival.

While this study has revealed an unexpected role for Atrx in the murine trophectoderm, as a result of the early lethality observed in Atrx^null^ embryos it is not possible to rule out other roles for Atrx at later developmental stages in tissues of the embryo proper. Indeed, we show that Atrx is highly expressed throughout the entire developing embryo at 7.5 dpc (Figure 5B), and it is likely that Atrx function will turn out to be important for other differentiating tissues. Tetraploid aggregation experiments (in which mutant embryos are rescued with wild-type extraembryonic tissues) might shed more light on the role of Atrx during later mouse development, but these issues can be more subtly dissected by combining the conditional *Atrx* ^flox^ allele that we have generated with different tissue-specific Cre transgenes. As mentioned above, this approach has already revealed a critical role for Atrx during neuronal differentiation in adult mice [20]. Further evidence that Atrx is also required at later stages of mouse development is provided by the observed dramatic skewing against Atrx-negative cells in some somatic tissues of carrier female mice, whose tissues initially comprise a mosaic of Atrx-positive and Atrx-negative cells as a result of random X-inactivation (M. Muers, personal communication).

*Atrx* joins an expanding list of mouse genes for which targeted disruption results in peri-implantation lethality as a result of trophoblast or placental abnormalities (reviewed in [25]). Comparison with other phenotypes might provide some insight into the role of Atrx in trophoblast development. *Atrx*-mutant embryos progress further than embryos nullizygous for factors involved in the initial specification of trophoblast stem cells (such as *Cdx2*) or in stem cell maintenance and proliferation (such as *Eomes*). *Cdx2*-mutant embryos fail to implant and die between 3.5 and 5.5 dpc [26], while *Eomes*-mutant blastocysts implant into the uterus, but arrest soon after implantation without forming organised embryonic or extraembryonic structures [27]. In contrast, *Atrx*-mutant embryos implant successfully and establish organised embryonic structures by 7.5 dpc. The *Atrx*-mutant phenotype closely resembles that observed in mice nullizygous for the basic helix-loop-helix transcription factor Hand1. *Hand1*-mutant conceptuses arrest at around 7.5 dpc and display a normal embryonic compartment, but, like *Atrx*-mutant embryos, ablation of Hand1 causes a reduction in the size of the ectoplacental cone and density of TGCs [28]. As with *Atrx* mutants, only arrested or resorbed *Hand1*-mutant conceptuses were recovered after 8.5 dpc. Also like Atrx, disruption of Hand1 specifically affects secondary giant cell formation, and primary trophoblast outgrowths from blastocysts appeared normal. Hand1 is required for terminal differentiation of secondary TGCs, and in its absence trophoblast cells arrest at a precursor stage in the ectoplacental cone [17,28]. Given the similarity of the *Atrx*- and *Hand1*-mutant phenotypes and the likelihood that Atrx acts as a transcriptional regulator by modifying chromatin structure, it will be of interest to determine whether Atrx is itself a regulator of *Hand1* expression, or alternatively whether it acts as a co-regulator of one or more of the downstream transcriptional targets of Hand1. It is noteworthy that, in the brain-specific *Atrx* knockout mice, the defect was observed in terminally differentiating neurons [20]. The secondary TGCs affected in the universal *Atrx* knockout reported here represent one of the first terminally differentiated tissues in the developing mouse, and this may point to the requirement for Atrx in the high-level expression of some tissue-specific genes during the final stages of differentiation. Interestingly, the α-globin genes, the only confirmed transcriptional targets of regulation by human ATRX, are also highly expressed specifically during terminal differentiation within the erythroid lineage.

### *Atrx* Escapes Imprinted X-Inactivation in Extraembryonic Tissues of Carrier Female Mice

Another surprising finding of this study is that, in carrier female embryos, a paternally inherited *Atrx* ^WT^ allele appears to escape the process of imprinted X-inactivation, which ordinarily silences the Xp chromosome in the extraembryonic compartment of female murine tissues [18]. Silencing of the *Atrx* ^WT^ allele on Xp should render these females null for *Atrx* in the extraembryonic tissues, since the normally active Xm chromosome carries the *Atrx* ^Δ18Δneo^ allele. Although not phenotypically normal, some *Atrx* carrier females developed to term and went on to reproduce. Thus, the failure to correctly silence the paternally derived *Atrx* ^WT^ allele in the extraembryonic tissues of carrier females is consistent with our observations that in Atrx^null^ males, the Atrx protein plays an essential role in the development of the trophoblast and is necessary for survival in utero in the mouse.

The survival of *Atrx* carrier females contrasts with the phenotypes seen in carriers of mutations of other murine X-linked genes known to be essential in the extraembryonic compartment. For example, targeted disruption of the dyskerin *(Dkc1),* glucose 6-phosphate dehydrogenase *(G6PD),* and choroideremia *(Chm)* genes cause embryonic lethality in null male embryos through defects of the extraembryonic-derived tissues [29–31]. Female mice carrying mutations of these genes on the maternally inherited X chromosome also die in utero, whereas females that inherit the mutation on the Xp chromosome survive. Thus, unlike *Atrx,* these genes and/or their effects on cell growth are unable to circumvent the processes that ultimately cause all cells in the extraembryonic tissues to express only the maternally derived X chromosome. How might expression of the paternal *Atrx* ^WT^ allele be maintained in the extraembryonic tissues of the *Atrx* carrier females?

One possibility is that, like some other X-linked genes, silencing of the *Atrx* gene on Xp is incomplete, such that there is always a low-level, leaky output of Atrx from a normally inactivated Xp chromosome in extraembryonic tissues. However, it was recently demonstrated that the paternal *Atrx* (called *Xnp*) allele is completely silenced in a normal mouse trophoblast stem cell line [32], suggesting that *Atrx* does not normally escape imprinted X-inactivation in the extraembryonic tissues of wild-type females. Thus, the expression of the Xp-linked *Atrx* ^WT^ allele that we observed is unique to female carriers of the *Atrx* ^null^ allele.

Perhaps a more likely explanation for this phenomenon stems from experimental observations suggesting that imprinted X-inactivation is not imposed on all precursors of the mouse extraembryonic tissues: A subpopulation of cells may escape this process and make a random “choice” of which X chromosome will be inactivated. On average, 50% of the cells in this randomly inactivating subpopulation would be expected to maintain an active Xp chromosome. In support of this hypothesis, it has been demonstrated that expression of paternally transmitted X-linked *lacZ* [33,34] and GFP [35] transgenes failed to be silenced in a small subpopulation of extraembryonic cells. Further, it has been shown that in a subpopulation of extraembryonic cells, it is the Xm rather than the Xp that undergoes late replication, a molecular correlate of the inactive state [18,36]. Although initially small and quickly diluted in normal embryos, the cellular subpopulation that inactivates the Xm chromosome could rapidly expand to replace the normally imprinted cells in extraembryonic lineages if the normal silencing of Xp compromises cell growth or differentiation. Interestingly, it has been suggested that the size of the population that initially escapes imprinting may range widely (from 0% to 30%), even between genetically identical embryos [37], and this may account for the variable phenotype observed among females bearing Xm-linked mutant alleles of genes essential for normal extraembryonic development [38]. Put simply, carrier females bearing a small initial population of escaping cells would be more severely affected than those bearing a larger population. This could explain why we have observed significant phenotypic variation among *Atrx* carrier females, with some carriers dying in utero by 9.5 dpc (Table 1) and others developing to term.

Another possible mechanism is that inactivation of the paternal X proceeds normally in all cells, but subsequently the *Atrx* gene within individual cells is reactivated. Alternatively, in the absence of Atrx, the paternal allele may partially escape the normal process of silencing. In both of these cases, other genes on the paternal X chromosome must be inactivated and remain so, since blocking inactivation of the entire Xp chromosome causes embryonic lethality due to biallelic expression of X-linked genes in the trophoblast [39].

### Summary

ATR-X syndrome is the first human genetic disease known to be caused by mutations in a chromatin remodelling factor. At present we do not know how ATRX influences gene expression or what effect it has on cell behaviour. Nevertheless, we have previously noted that none of the natural mutations causing ATR-X syndrome are nulls, which suggests that it plays a critical role in normal development. Results of conditional inactivation of Atrx in the developing mouse forebrain, based on the *Atrx* ^flox^ allele described here, shows that Atrx exerts a major effect on terminally differentiating neurons. Conditional inactivation of Atrx in other tissues is underway. Here we have shown that animal-wide disruption of the *Atrx* gene causes a severe embryonic-lethal phenotype, revealing an essential role for Atrx in the formation of the murine trophoblast. In addition, Atrx appears to escape imprinted X-chromosome inactivation in the extraembryonic tissues of some carrier female mice.

## Materials and Methods

### Generation of ES cells bearing the *Atrx* ^flox^ allele.

Briefly, the targeting vector (shown in Figure 2A) places a *loxP* site within intron 18 and a *loxP*-flanked MC1neopA selection cassette in intron 17 of the *Atrx* gene. A detailed description of the targeting construct is provided in [20]. Linearised plasmid (150 μg) was electroporated into 1 × 10^8^ E14Tg2a ES cells, and colonies resistant to G418 and ganciclovir were isolated. Homologous targeting events were identified by Southern blot of EcoRI-digested DNA and hybridisation with a 5′ probe (generated with primers PPS1.20 and PPS1.27) and a 3′ probe (a 0.9-kb HaeIII fragment) as shown in Figure 2A and 2B. DNA from correctly targeted clones was also digested with SacI and analysed by Southern blot and hybridisation with a probe from within intron 17 (a PCR product generated with primers PPS1.15 and Xnp46) to confirm that the *loxP* site within intron 18 had been included within the crossed-over region (Figure 2A and 2C). Sequences of primers are shown in Table S1.

### Cre-recombination and characterisation of Atrx^null^ ES cells and embryoid bodies.

ES cell clones bearing the *Atrx* ^flox^ allele (1 × 10^7^ cells) were transiently transfected with 50 μg of uncut Cre expression plasmid (pCAGGS-Cre-IRES*puro*) [40]. Following transfection, cells were plated at a range of clonal densities in complete medium without G418, and isolated subclones were picked after 7 d. Subclones were expanded and analysed for the presence of a recombinant locus initially by PCR, to detect deletion of the MC1neopA cassette, and then by Southern blot and hybridisation with the intron 17 probe described above (Figure 2A and 2C). Northern blots were carried out according to standard techniques using 20 μg of total RNA isolated using TRI Reagent (Sigma-Aldrich, St. Louis, Missouri, United States). The blot was hybridised with a probe from within exon 10 of the *Atrx* gene (generated with primers Mxnp4 and Mxnp28 [Table S1]). After it was stripped, the membrane was hybridised with a β-actin cDNA probe (Clontech, Palo Alto, California, United States). Protein extraction and detection of Atrx by Western blotting was performed as described previously [4], using the mouse monoclonal anti-ATRX antibody 23C [15]. Analyses of cell cycle and apoptosis are described in Protocol S1. Methylation of rDNA was analysed in DNA from ES cell clones or from embryoid bodies recovered after 7 d of in vitro differentiation as described previously [41]. Genomic DNA was digested with methylation-sensitive restriction enzymes as described and analysed by Southern blotting. The RIB3 and RIB4 probes (which were amplified from human DNA, but cross-react with the mouse rDNA repeat) have been described previously [11]. Oligonucleotide probes to detect Line 1 and Sine B1 repeats have been described previously [42]. The minor satellite probe was a 27-mer oligonucleotide (mCENT2). The major satellite probe was a 27-mer oligonucleotide (DG27). The IAP probe was an ~400 bp PCR product (primers 14A and 13K) amplified from an IAP inserted into the mouse *agouti* gene [43] and was a gift from Peter Warnecke and Tim Bestor. The PCR product included the entire 5′ LTR of the IAP. All oligonucleotide sequences are shown in Table S1.

### Generation of chimeras, floxed mice, and mutant mice.

Targeted *Atrx* ^flox^ ES cell clones were injected into C57BL/6 blastocysts and transferred into 2.5 dpc pseudopregnant recipients by standard techniques. Resulting chimeras were mated with C57BL/6 to establish germline transmission. Offspring with the *Atrx* ^flox^ allele were identified by Southern blotting of SacI-digested tail DNA using the intron 17 probe as shown in Figure 2A and 2C. For Cre-recombination, *Atrx* ^flox^ mice were crossed with *GATA1-Cre* transgenic mice as described in the text. Recombinant alleles were detected by Southern blotting as described above or by PCR as described in Protocol S1.

### Immunohistochemistry, in situ hybridisation, and TUNEL assay.

7.5 dpc decidual swellings were dissected away from maternal tissue and fixed in 4% paraformaldehyde/PBS overnight at 4 °C. After embryos were washed thoroughly in PBS, they were dehydrated through an ethanol series and xylene, embedded in paraffin, and sectioned at 5 μm. Sections were processed for immunohistochemistry using the ABC Staining System (Santa Cruz Biotechnology, Santa Cruz, California, United States) according to the manufacturer's instructions. Sections were stained with rabbit polyclonal antibodies against ATRX (H-300, Santa Cruz Biotechnology), Placental lactogen-I (AB1288, Chemicon International, Temecula, California, United States) and phospho (Ser10)-histone H3 (06–570, Upstate Biotechnology, Waltham, Massachusetts, United States). Where appropriate, adjacent sections were stained with haematoxylin. In some cases, adjacent sections were also analysed to detect apoptotic cells by TUNEL using the in situ cell death detection kit (Roche, Basel, Switzerland). After labelling, these slides were mounted in Vectashield containing DAPI (Vector Laboratories, Burlingame, California, United States) and visualised by fluorescence microscopy. Whole-mount in situ hybridisations were performed on 8.5 dpc embryos (dissected away from maternal and extraembryonic tissues) using a *brachyury (T)* riboprobe [16] and on bisected decidual implantation sites from which embryos (8.5 dpc) had been removed using a placental lactogen-1 (*Pl-1*) riboprobe (see Protocol S1 for details).

### Blastocyst outgrowth cultures.

Superovulated heterozygous female mice (*Atrx* ^WT/flox^) were mated to homozygous *GATA1-cre* ^+/+^ transgenic males, and blastocysts were flushed from uterine horns with M2 medium (Sigma) at 3.5 dpc. Individual blastocysts were cultured in multiwell tissue cultures plates as described previously [44]. Cultures were inspected and photographed daily and the extent of outgrowth scored. After 7 d, cultures were harvested and DNA extracted. The *Atrx* genotype and sex of each culture was determined by PCR as described in Protocol S1.

## Supporting Information

Figure S1 Cell Cycle Analysis of Atrx-Positive and Atrx^null^ ES Cells (A) Representative FACS profiles of BrdU-pulsed ES cells bearing either functional (*Atrx* ^WT^ or *Atrx* ^flox^) or null (*Atrx* ^Δ18Δneo^) *Atrx* alleles showing BrdU-FITC (*y*-axis, logarithmic scale) against propidium iodide (*x*-axis, linear scale). The gated populations show cells in G1 (PI^low^, BrdU-negative) (R1), S (BrdU-positive) (R2), and G2/M (PI^hi^, BrdU-negative) (R3) phases of the cell cycle. Below is shown the quantitation of gated populations, indicating the percentage of cells in G1, S, and G2/M cell-cycle phases in each culture.
(B) FACS profiles of ES cells bearing either functional (*Atrx* ^WT^ or *Atrx* ^flox^) or null (*Atrx* ^Δ18Δneo^) *Atrx* alleles stained for the mitosis marker phosphorylated (Ser10) histone H3 (FITC, *y*-axis, logarithmic scale) against propidium iodide (*x*-axis, linear scale). The size of the mitotic population (phosphoH3S10-positive, PI^hi^)(gate R3) is indicated for each profile. The ES cell clone analysed in each trace is indicated.
(3.2 MB PDF)

Figure S2 Analysis of Apoptosis in Atrx-Positive and Atrx^null^ ES Cells FACS analysis of ES cells bearing different *Atrx* alleles as shown, after costaining for Annexin V (FITC, *x*-axis, logarithmic scale) and propidium iodide (*y*-axis, logarithmic scale). The size of the early apoptotic (Annexin-positive, PI^low^) and late apoptotic or necrotic (Annexin-positive, PI^hi^) populations is indicated for each genotype.
(223 KB PDF)

Figure S3 Normal DNA Methylation at Mouse Repetitive Elements in Atrx^null^ ES Cells DNA from Atrx-positive (bearing either an *Atrx* ^WT^ or *Atrx* ^flox^ allele) or Atrx^null^ (bearing the *Atrx* ^Δ18Δneo^ allele) ES cells was digested with either a CpG-methylation-sensitive enzyme HpaII (H) or its methylation-insensitive isoschizomer MspI (M) as indicated, and digested DNA was analysed by Southern blotting. Membranes were hybridised with probes specific for the mouse Line 1 (A), Sine B1 (B), minor satellite (C), and IAP (D) repeat elements. No significant loss of CpG-methylation was observed at any of these repetitive elements in Atrx^null^ ES cells. As a positive control for loss of methylation, DNA from ES cells lacking either the Dnmt1 (Dnmt1^−/−^) or both the Dnmt3a and Dnmt3b (Dnmt3a3b^−/−^) DNA methyltransferases was included in the analysis of Line 1 and Sine B1 repeats. Loss of CpG-methylation was clearly observed at these repetitive elements in these cell lines.
(1.8 MB PDF)

Figure S4 Escape from Imprinted Inactivation in 8.5 dpc Carrier Female Embryo A cross was carried out between a carrier female (*Atrx* ^WT/null^) and wild-type male (*Atrx* ^WT^/Y), and embryos were dissected in their deciduas at 8.5 dpc. Any carrier female progeny of this cross will carry an *Atrx* ^WT^ allele on the Xp chromosome and an *Atrx* ^null^ allele on the Xm chromosome. Transverse paraffin sections were stained with the anti-ATRX antibody (H-300).
(A) High-magnification image (400×) of the neural fold region (nf) (embryo proper) of a carrier female (*Atrx* ^WT/null^) embryo. This tissue is clearly comprised of a mosaic of Atrx-positive and Atrx-negative cells due to random X-inactivation. This section was counterstained with haematoxylin.
(B) High-magnification image (400×) of the same embryo depicted in (A), showing the extraembryonic derived-chorionic ectoderm (ce) and visceral endoderm (ve), two tissues that undergo imprinted X-inactivation. Although Atrx is poorly expressed in the visceral endoderm (even in wild-type females [unpublished data]), strong expression of Atrx can be seen in the chorionic ectoderm, indicating that the paternally-derived *Atrx* ^WT^ allele had escaped inactivation in this tissue.
(1.7 MB PDF)

Protocol S1 Supplementary Methods (42 KB DOC)

Table S1 Primers Used in This Study (20 KB DOC)

### Accession Numbers

The Online Mendelian Inheritance in Man (http://www.ncbi.nlm.nih.gov/entrez/query.fcgi?db=OMIM) accession number for ATR-X syndrome is 301040. The GeneID (www.ncbi.nlm.nih.gov/entrez/query.fcgi?db=gene) for human *ATRX* is 546 and for mouse *Atrx* is 22589. The GenBank (http://www.ncbi.nlm.nih.gov/) accession number for the minor satellite probe mCENT2 is X14470 (nucleotides 75–101), for the major satellite probe DG27 is M25032 (nucleotides 146–172), and for the IAP probe is L33247.

## Abbreviations

BrdU: bromodeoxyuridine
dpc: days postcoitus
ES cells: embryonic stem cells
IAP: intracisternal A particle
rDNA: ribosomal DNA
TGC: trophoblast giant cell
TUNEL: TdT-mediated dUTP nick end labeling
WMISH: whole-mount in situ hybridisation
