# Supplementary material for: Loss of Atrx Affects Trophoblast Development and the Pattern of X-Inactivation in Extraembryonic Tissues
Source: PLoS Genet. 2006 Apr 21;2(4):e58. doi: 10.1371/journal.pgen.0020058 (PMC1440874; doi:10.1371/journal.pgen.0020058)
Supplement: Supplementary file 1 [file pgen.0020058.sg001.pdf]

A

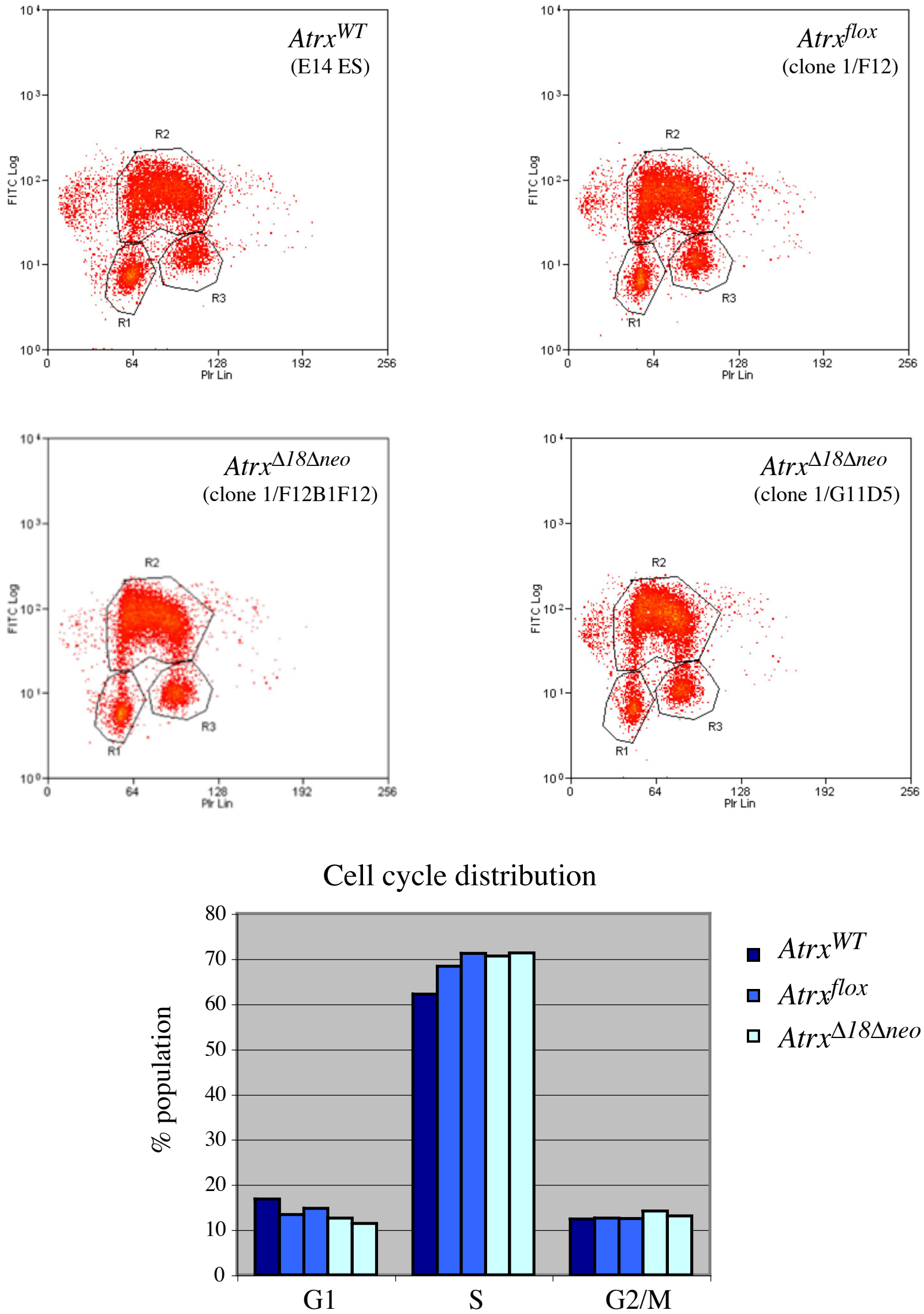
AtrxWT
(E14 ES)
Atrxflox
(clone 1/F12)
AtrxΔ18Δneo
(clone 1/F12B1F12)
AtrxΔ18Δneo
(clone 1/G11D5)
R1
R2
R3
FITC Log
Plr Lin
Cell cycle distribution
% population
G1
S
G2/M
AtrxWT
Atrxflox
AtrxΔ18Δneo

B

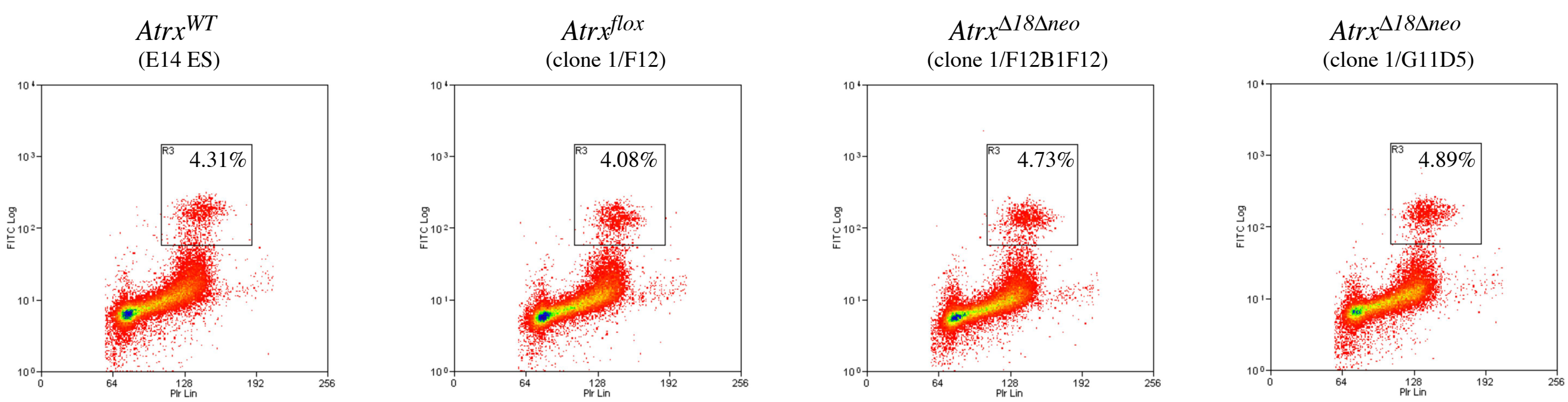
AtrxWT
(E14 ES)
Atrxflox
(clone 1/F12)
AtrxΔ18Δneo
(clone 1/F12B1F12)
AtrxΔ18Δneo
(clone 1/G11D5)
4.31%
4.08%
4.73%
4.89%
R3
FITC Log
Plr Lin
