# Supplementary material for: Loss of Atrx Affects Trophoblast Development and the Pattern of X-Inactivation in Extraembryonic Tissues
Source: PLoS Genet. 2006 Apr 21;2(4):e58. doi: 10.1371/journal.pgen.0020058 (PMC1440874; doi:10.1371/journal.pgen.0020058)
Supplement: Supplementary file 2 [file pgen.0020058.sg002.pdf]

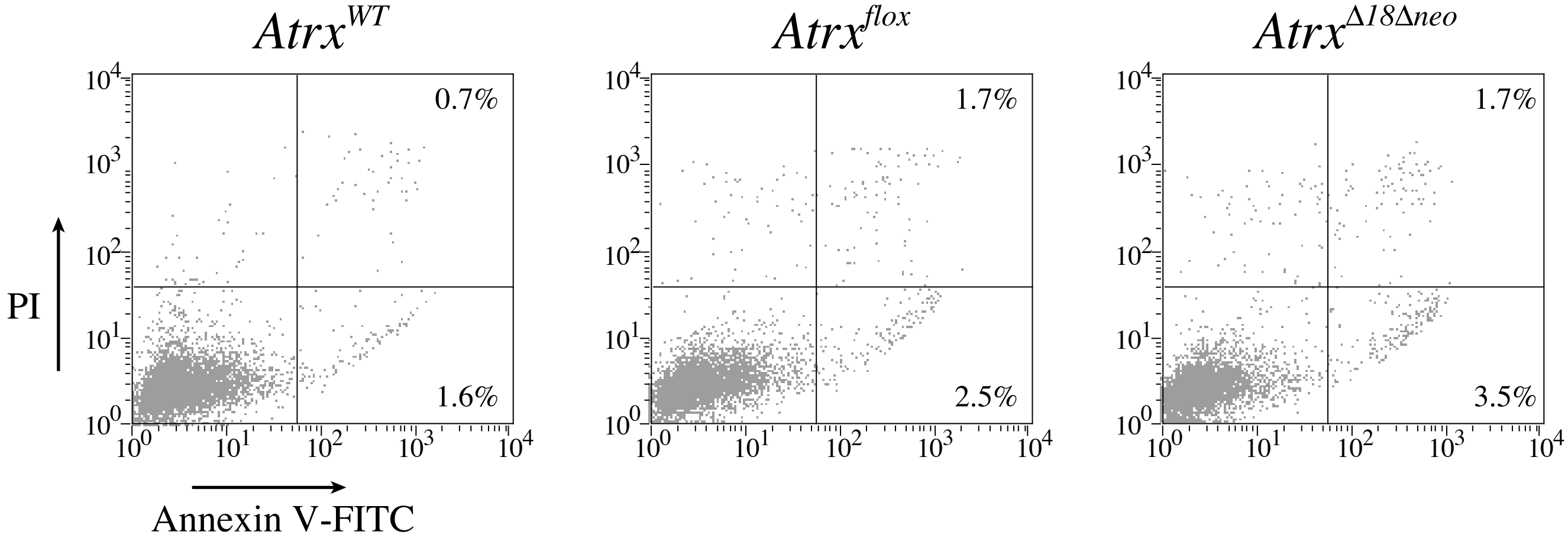

Atrx^WT
Atrx^flox
Atrx^Δ18Δneo
0.7%
1.6%
1.7%
2.5%
1.7%
3.5%
PI
Annexin V-FITC
