# Supplementary material for: Loss of Atrx Affects Trophoblast Development and the Pattern of X-Inactivation in Extraembryonic Tissues
Source: PLoS Genet. 2006 Apr 21;2(4):e58. doi: 10.1371/journal.pgen.0020058 (PMC1440874; doi:10.1371/journal.pgen.0020058)
Supplement: Supplementary file 3 [file pgen.0020058.sg003.pdf]

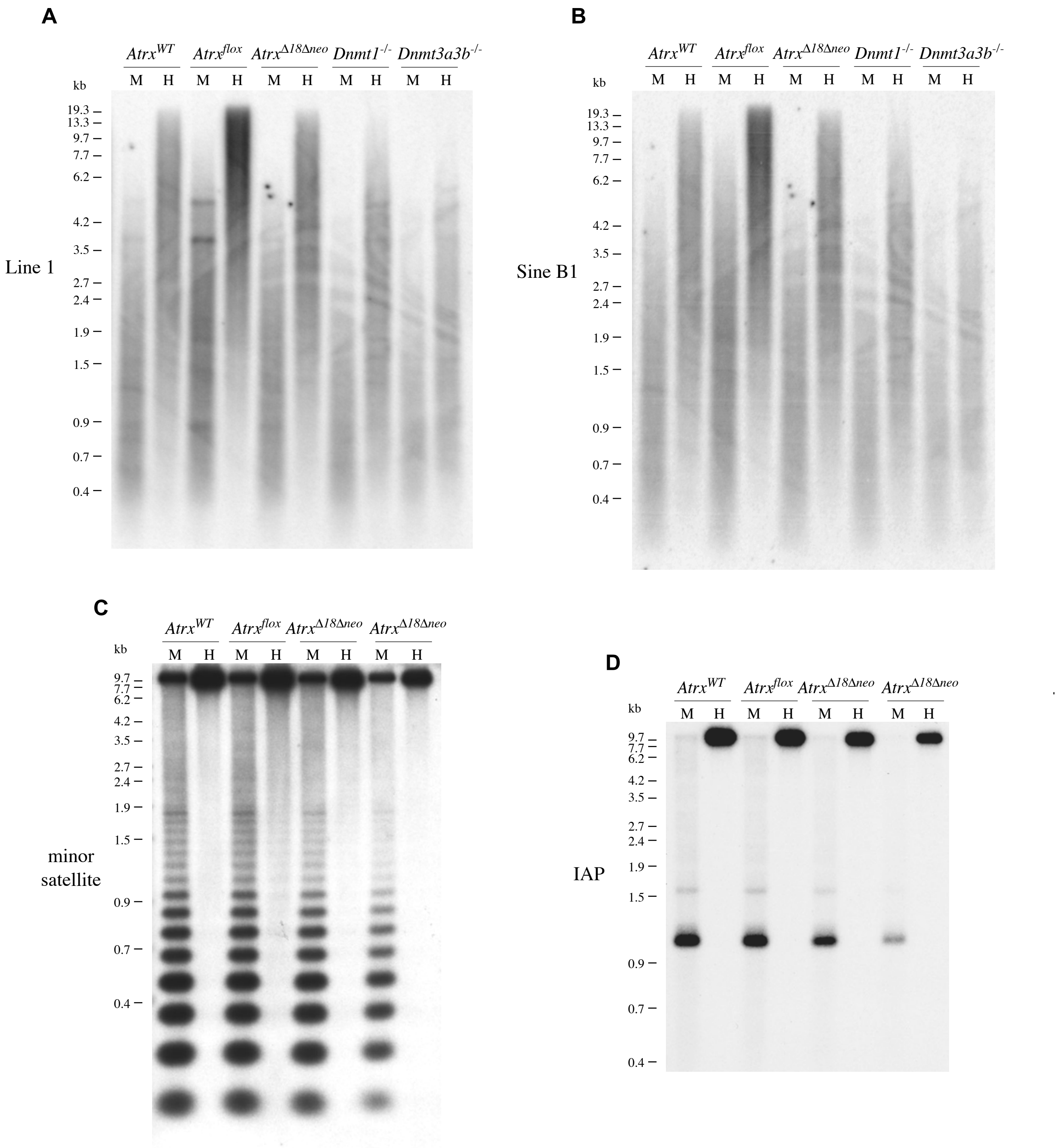
A
AtrxWT
Atrxflox
AtrxΔ18Δneo
Dnmt1-/-
Dnmt3a3b-/-
M H M H M H M H M H
kb
19.3
13.3
9.7
7.7
6.2
4.2
3.5
2.7
2.4
1.9
1.5
0.9
0.7
0.4
Line 1
B
AtrxWT
Atrxflox
AtrxΔ18Δneo
Dnmt1-/-
Dnmt3a3b-/-
M H M H M H M H M H
kb
19.3
13.3
9.7
7.7
6.2
4.2
3.5
2.7
2.4
1.9
1.5
0.9
0.7
0.4
Sine B1
C
AtrxWT
Atrxflox
AtrxΔ18Δneo
AtrxΔ18Δneo
M H M H M H M H
kb
9.7
7.7
6.2
4.2
3.5
2.7
2.4
1.9
1.5
0.9
0.7
0.4
minor satellite
D
AtrxWT
Atrxflox
AtrxΔ18Δneo
AtrxΔ18Δneo
M H M H M H M H
kb
9.7
7.7
6.2
4.2
3.5
2.7
2.4
1.9
1.5
0.9
0.7
0.4
IAP
