# Supplementary material for: Loss of Atrx Affects Trophoblast Development and the Pattern of X-Inactivation in Extraembryonic Tissues
Source: PLoS Genet. 2006 Apr 21;2(4):e58. doi: 10.1371/journal.pgen.0020058 (PMC1440874; doi:10.1371/journal.pgen.0020058)

Atrx$^{wt/null}$ carrier female (8.5 dpc)

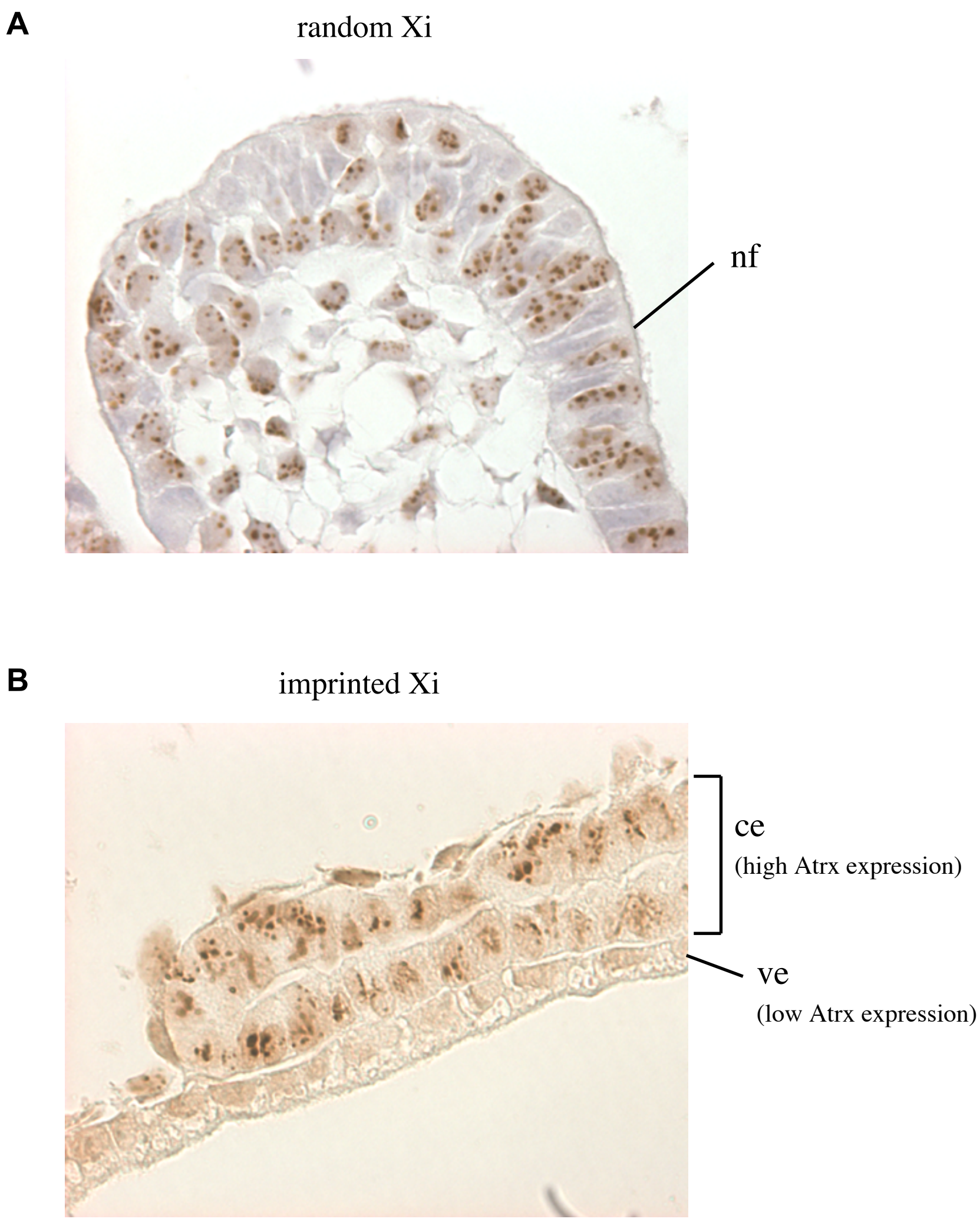

Supplement: Supplementary file 4 [file pgen.0020058.sg004.pdf]
