# Supplementary material for: Loss of Atrx Affects Trophoblast Development and the Pattern of X-Inactivation in Extraembryonic Tissues
Source: PLoS Genet. 2006 Apr 21;2(4):e58. doi: 10.1371/journal.pgen.0020058 (PMC1440874; doi:10.1371/journal.pgen.0020058)
Supplement: Supplementary file 5 [file pgen.0020058.sd001.doc]

## Supplementary Methods

### Cell cycle and apoptosis analysis of ES cells

For cell cycle analysis, ES cells (~50% confluent) were pulsed with 30 μM BrdU (Sigma) at 37°C for 30 min. Cells were harvested, washed in PBS and fixed in cold 70% ethanol at 4°C overnight. Prior to staining, cells were washed twice in PBS and then histones were extracted by incubating in 2M HCl for 30 min at room temperature. Cells were washed twice in PBS and once in PBS / 0.1% BSA / 0.2% Tween-20 (PBS-T). Cells were incubated for 30 min at room temperature with a mouse monoclonal anti-BrdU antibody (MD5010, Caltag), washed twice in PBS-T, and then incubated for 30 min at room temperature with an Alexa488-conjugated donkey-anti-mouse secondary antibody (A21202, Molecular Probes). After washing twice in PBS-T, cells were resuspended in propidium iodide (PI) staining solution (40 μg/ml PI and 100 μg/ml RNaseA in PBS), incubated in the dark for 15 min at 37°C and allowed to equilibrate at 4°C in the dark overnight. For the detection of mitotic cells, fixed ES cells from above were stained with an anti-phospho (Ser10)-histone H3 primary antibody (06-570, Upstate Biotechnology) and an Alexa488-conjugated goat-anti-rabbit secondary antibody (A11034, Molecular Probes), essentially as described in [1]. For both analyses, stained cells were analysed on a CyanADP flow cytometer using Summit v4.1 software (DakoCytomation). Cell doublets (detected by pulse-width signal) were gated out.

Analysis of apoptosis was carried out in ES cells using the TACS Annexin V-FITC kit (R&D Systems) according to the manufacturer’s instructions. Cells were analysed on a FACSCalibur flow cytometer using CellQuest software (Becton Dickinson).

## *Genotyping embryos and blastocyst outgrowth cultures by PCR*

DNA was extracted from yolk sac or total embryos at 7.5, 8.5 and 9.5 dpc or *in vitro* blastocyst cultures. Embryonic tissues were digested in TE containing 0.1% Tween 20 and proteinase K (0.1 mg/ml) at 55°C for 2 hours. For blastocyst outgrowths, 7 day cultures were lysed in 100 μl cell lysis buffer (10 mM Tris pH 8.0, 10 mM NaCl, 10 mM EDTA, 0.5% SDS, 50 μg/ml proteinase K) and digested at 37°C overnight. DNA was extracted in phenol:chloroform (1:1) and chloroform, and then ethanol precipitated using 20 μg yeast tRNA as carrier. Pellets were typically resuspended in 10-25 μl Milli-Q water and 1 μl used in PCR reactions. Two different PCR reactions were used to determine the *Atrx* genotype:

a) Primers PPS1.15 (exon 17) and Mxnp30 (exon 20): A 25 μl reaction containing 0.4 μM each primer, 0.2 mM each deoxynucleotide triphosphate and 0.9 U Expand High Fidelity Enzyme (Roche) in 1X Expand buffer with 1.5 mM MgCl_2_was cycled as follows: 94°C for 2 min followed by 10 cycles of 94°C for 15 s, 55°C for 30 s, 72°C for 2 min 30 s followed by 24-26 cycles in which the extension time was increased by 5 s / cycle. The expected allele sizes for this PCR are: 3.2 kb for *Atrx^flox^*, 2.0 kb for *Atrx^WT^*, 2.2 kb for *Atrx^Δ18^* and 1.1 kb for *Atrx^Δ18Δneo^*. The last two alleles (resulting from recombination events B and C in Fig.2A) are both null for full length Atrx. The results of this PCR are shown in Fig.4B.

b) Primers PPS1.17 (intron 17) and PPS1.28 (exon 19): A 25 μl reaction containing 0.4 μM each primer, 0.2 mM each deoxynucleotide triphosphate and 1.25 U Taq (Roche) in 1X supplied buffer with 1.5 mM MgCl_2_ was cycled as follows: 94°C for 2 min followed by 30-32 cycles of 94°C for 30 s, 55°C for 30 s, 72°C for 1 min. The expected allele sizes for this PCR are: 1.4 kb for *Atrx^WT^*, 1.6 kb for *Atrx^Δ18^* and 0.6 kb for *Atrx^Δ18Δneo^*. The last two alleles (resulting from recombination events B and C in Fig.2A) are both null for full length Atrx. The *Atrx^flox^* allele is not detected in this reaction.

To determine sex, primers DG52 & DG53 were used to amplify a 450 bp product from a mouse Y-chromosome-specific satellite repeat as follows: A 25 μl reaction containing 0.3 μM each primer, 0.2 mM each deoxynucleotide triphosphate and 1.25 U Taq (Roche) in 1X supplied buffer with 1.5 mM MgCl_2_ was cycled as follows: 95°C for 2 min followed by 29-30 cycles of 94°C for 30 s, 60°C for 30 s, 72°C for 1 min. The results of this PCR are shown in Fig.4B. All primer sequences are shown in Supplementary Table I.

#### Synthesis of mouse Pl-1 riboprobe and whole mount in situ hybridization

To generate the *Pl-1* riboprobe, a ~600 bp *Hin*dIII / *Pst*I fragment from pRSV-mPL-1 [2] containing bases 1-582 of the mouse placental lactogen-1 mRNA (Genbank accession number M35662) was subcloned into corresponding sites of pGEM7Zf(+) (Promega). The plasmid was linearised with *Eco*RI and an antisense *Pl-1* riboprobe was generated using SP6 RNA polymerase. For whole mount *in situ* hybridization, tissues were fixed in 4% paraformaldehyde / PBS overnight at 4°C and washed in several changes of cold PBS. WMISH was carried out according to [3] using hybridization conditions of [4]. Tissues were processed for photography through a glycerol series (50, 80, and 100%) and photographed in 100% glycerol on a glass slide.

## Supplementary References

1. Xu B, Kim S, Kastan MB (2001) Involvement of Brca1 in S-phase and G(2)-phase checkpoints after ionizing irradiation. Mol Cell Biol 21: 3445-3450.

2. Colosi P, Talamantes F, Linzer DI (1987) Molecular cloning and expression of mouse placental lactogen I complementary deoxyribonucleic acid. Mol Endocrinol 1: 767-776.

3. Wilkinson DG (1992) Whole mount in situ hybridisation of vertebrate embryos. In: Wilkinson DG, editor. In situ Hybridisation Practical Approach Series. Oxford: IRL Press. pp. 75-83.

4. Rosen B, Beddington RS (1993) Whole-mount in situ hybridization in the mouse embryo: gene expression in three dimensions. Trends Genet 9: 162-167.
