# Supplementary material for: Loss of Atrx Affects Trophoblast Development and the Pattern of X-Inactivation in Extraembryonic Tissues
Source: PLoS Genet. 2006 Apr 21;2(4):e58. doi: 10.1371/journal.pgen.0020058 (PMC1440874; doi:10.1371/journal.pgen.0020058)
Supplement: Supplementary file 6 [file pgen.0020058.st001.doc]

# Supplementary Table I. Primers used in this study

14A	5’NNNNGGGCCCGCAGAGAAGGTGATCGGTGG

13K	5’NNNNGGTACCTGTTATTCGACGCGTTCTCA

DG27	5’TCATGGAAAATGAGAAACATCCACTTG

DG52	5’TGACGTTTTCGCCTCGGGAC

DG53	5’GGGAGGAGTTCTGGAATACCACTTC

mCENT2	5’CTCATGATATACACTGTCTACAATGCC

Mxnp4	5’GCTTCCTTTTAGGAGTTGCTC

Mxnp28	5’GGAACAAAAGGAAAGAAAGCCC

Mxnp30	5’CACCATCTTCTTGCCATCTCTGTAG

PPS1.15	5’GGTTTTAGATGAAAATGAAGAG

PPS1.17	5’AACTCATTCAACTGCCC

PPS1.20	5’CAGGAGTCAGGAGTTAG

PPS1.27	5’ CAGCTTTAGTGGTTTGTCCTC

PPS1.28	5’ CATTTAATCCCTCCTGCC

Xnp46	5’ACAGACTCACAGCAGCAATCCCAC
